# Effectiveness of implementing of an infection control link nurse program to improve compliance with standard precautions and hand hygiene among nurses: a quasi-experimental study

**DOI:** 10.1186/s12909-023-04208-1

**Published:** 2023-04-19

**Authors:** Shamsi Ghorbanmovahhed, Shahla Shahbazi, Neda Gilani, Ali Ostadi, Reza Shabanloei, Leila Gholizadeh

**Affiliations:** 1 Department of Medical-Surgical Nursing, Faculty of Nursing and Midwifery, Tabriz University of Medical Sciences, Tabriz, Iran; 2 Department of Medical-Surgical Nursing, Faculty of Nursing and Midwifery, Tabriz University of Medical Sciences, PO Box 5138947-977, Tabriz, Iran; 3 Clinical Research Development Unit, Sina Educational, Research and Treatment Center, Tabriz University of Medical Sciences, Tabriz, Iran; 4 Department of Statistics and Epidemiology, Faculty of Health, Tabriz University of Medical Sciences, Tabriz, Iran; 5 Department of Internal Medicine, Sina Educational, Research and Treatment Center, faculty of Medicine, Tabriz University of Medical Sciences, Tabriz, Iran; 6 Faculty of Health, University of Technology Sydney, Sydney, Australia

**Keywords:** Hand hygiene, Standard precautions, Compliance, Infection control link nurse program, Healthcare-associated infections, Nurse

## Abstract

**Background:**

Standard precautions (SPs) including hand hygiene are considered fundamental protective measures to manage health care-associated infections (HCAIs) and to reduce occupational health hazards. The purpose of this research was to examine the effectiveness of an infection control link nurse (ICLN) program on compliance with SPs and hand hygiene among nurses.

**Methods:**

A quasi-experimental study with a pretest-post-test design was conducted with participating of 154 clinical nurses who worked in different wards of a tertiary referral teaching hospital in Iran. The intervention group (n = 77) had 16 infection control link nurses nominated. The control group (n = 77) received only the standard multimodal approach used in the hospital. Pre- and post-test assessment of compliance with standard precautions and hand hygiene compliance was performed via the Compliance with Standard Precautions Scale (CSPS) and the World Health Organization observational hand hygiene form. Two independent sample t-tests were used to examine differences between Compliance with Standard Precautions and hand hygiene Compliance among nurses in intervention and control group. Multiple linear regression analysis was used to assess the effect size.

**Results:**

After developing and implementing the infection control link nurse program, no statistically significant improvement was found in the Compliance with Standard Precautions (β = 5.18; 95% CI= -0.3–10.65, p = 0.064). An improvement in hand hygiene compliance was observed among nurses in the intervention group that improved statistically significant from 18.80% before the program to 37.32% 6 months after the program (β = 20.82; 95% CI 16.40–25.25, p < 0.001).

**Conclusions:**

Given the continuing level of interest that exists in improving health care workers’ hand hygiene practices, the findings of this study provide significant practical implications for hospitals seeking to improve compliance with hand hygiene among nurses, showing the effectiveness of using infection control link nurse program. Further research is needed to assess effectiveness of using infection control link nurse program to improve compliance with standard precautions.

## Background

Health care–associated infections (HCAIs) contribute to significant morbidity and mortality, particularly in low- and middle-income countries [1]. Infection prevention and control (IPC) addresses the spread of infections from patient to patient, patients to staff, staff to patients, or among staff within health care systems and includes prevention and monitoring strategies, such as hand hygiene, cleaning/disinfection/sterilization, vaccination, and surveillance, monitoring/investigation of demonstrated or suspected spread of infection within a particular health care setting as well as management of outbreaks [2]. About 10–70% of HCAIs are preventable [3]. Compliance with standard precautions is a simple and effective approach in prevention of HCAIs. Nevertheless, compliance with standard precaution measures is still suboptimal among health care workers (HCWs) [4].

Based on World Health Organization (WHO), health care workers’ hand hygiene plays a critical role in patient safety [5]. HCWs can spread infection-causing microorganisms if they do not perform hand hygiene at key moments using effective methods. Hand hygiene reduces transmission of microorganisms including those that are antibiotic-resistant, decreases HCAIs, and improves patient safety [6].

Strategies to improve compliance with standard precautions and hand hygiene require leadership, commitment and resourcing. The leadership should promote compliance with standard precautions and hand hygiene as an organizational priority and reinforce hand hygiene behaviour through role-modelling [7, 8].

Interventions to increase healthcare workers’ compliance with standard precautions and hand hygiene include but not limited to using a multifaceted set of interventions [9], implementing the WHO’s Multimodal Hand Hygiene Improvement Strategy [10], hand hygiene role modelling [11], in-service training about hand hygiene [12], and implementing infection control link nurse **(**ICLN**)** programs [13]. The ICLN programs aim to increase health care workers’ understanding of infection prevention, create a liaison between hospital wards and the IPC team, and to promote ICLN as a source of information for their peers [14].

Early research has explored a range of benefits from implementing ICLN programs to improve compliance and strengthen IPC measures [8, 13]. Sopirala et al. (2014) reported that the ICLN program was effective in reducing HCAIs including methicillin-resistant Staphylococcus aureus infections [15]. However, robust evidence is lacking on the effectiveness of these programs to improve compliance with standard precautions and hand hygiene [13, 14]. Although the ICLN programs have been implemented in many health care systems in developed countries [14], it is a new concept to the health care system of Iran. Available evidence suggests that HCIs occur at a high rate in Iran [16], nevertheless, Seifi et al. (2019) stated that the HCIs cases were not reported accurately in Iran [17]. They suggested that the implementation of ICLN programs might help improve the Nosocomial Infection Surveillance System in this country through facilitating accurate collection and report of HCAIs data. This study aimed to examine the effectiveness of an ICLN program to improve compliance with standard precautions and hand hygiene among nurses.

## Methods

### Research design

This research used a quasi-experimental design. Quasi-experimental designs facilitate the examination of causality in situations in which a complete control of the research setting is not possible [18]. These designs aim to control as many threats to validity as possible in a situation in which at least one of the three components of true experimental design including randomization, comparison of groups, and controlled manipulation of the treatment is lacking [18].

### Study setting

The setting of this study was Sina Educational, Research and Treatment Center. This is a teaching referral hospital in the northwest of Iran. The facility has an IPC team to address HCAIs.

### Randomization

The study used cluster randomization to randomly assign 16 medical-surgical wards and intensive care units to the study groups. First, the hospital wards were divided into two matching groups in terms of type of ward, type of patients, and nursing care provided. The groups were then randomly allocated to intervention group (the ICLN program) or control. An overall 154 nurses from the 16 participating wards were involved in the study, 77 nurse participants in each study group. To be included in the study, nurses needed to be working as a floor nurse providing direct care to patients, and to consent to participate in the research.

### Outcome measures

The primary outcome was compliance with standard precautions and hand hygiene.

### Definitions of terms

**Standard precautions** refer to a system of actions that applies to all patients, regardless of their presumed or confirmed infectious status. Standard precautions represent the primary strategy for preventing HCAIs. They include but are not limited to hand hygiene, the use of personal protective equipment (PPE), proper handling of patient care equipment and linen, environmental control, prevention of injury from sharp devices, correct waste disposal, and correct management of used needles and other sharp objects [19]. The definitions of the key concepts were adopted from the WHO’s Hand Hygiene Technical Reference Manual [20]; **hand hygiene** was defined as a general term referring to a hand cleansing using an alcohol-based hand rub or handwashing with water and soap with the aim of aimed at reducing or inhibiting the growth of micro-organisms on hands [20]; a **hand hygiene opportunity** was defined as a moment during healthcare activities when hand hygiene is required, regardless of the number of indications. **Indications** (five indications) were defined as before touching a patient, before a clean/aseptic procedure, after body fluid exposure risk, after touching a patient, after touching patient surroundings. Several indications may arise simultaneously, creating a single opportunity and requiring a single hand hygiene action [20]. **Compliance with hand hygiene** was defined as the observable behaviour of nurses following the guidelines for hand hygiene in the care of all patients [21]. Hand hygiene compliance was calculated by dividing the number of performed hand hygiene moments by the number of hand hygiene opportunities [21]. **The infection control link nurse (ICLN)** in this study was an experienced nurse interested in infection prevention and control, who was selected via self- nomination or nomination by the nurse unit manager. ICLN acted as a liaison between their colleagues in ward and the ICP team in the hospital. They contributed to raising the awareness of infection prevention and control among other nurses and promoting infection control practices in the workplace.

### Data collection

Data were collected using the Compliance with Standard Precautions Scale (CSPS) [22], and Hand Hygiene Audit Checklist [5, 20].

### Compliance with Standard Precautions Scale (CSPS)

The CSPS is a widely used self- report scale that assesses nurses’ level of compliance with standard precautions. It consists of 20 items and five dimensions, including compliance with the use of personal protective equipment, disposal of sharps and wastes, decontamination of spills and used articles, and prevention of cross infection. The prevention of cross infection from person to person dimension contains of 7 items, of which 5 items (Item 1, Item 2, Item 3, Item 11, and Item 12) are assessing some aspects of hand hygiene compliance. The scale uses a 4-point Likert type scale with response options ranging from never to always. ‘Always’ responses are scored one and ‘sometimes’, ‘rarely’, and ‘never’ are scored zero. For reverse items (items 2, 4, 6, and 15), ‘never’ responses are scored one and the remaining zero. Individual item scores are summed up to compute the total score, which can range from 0 to 20, with higher values indicating a better compliance [22, 23]. The CSPS has adequate psychometric properties to measure nurses’ compliance with standard precautions [13, 23, 24].

### Hand Hygiene Audit Checklist

Internationally, health care workers’ hand hygiene practices are guided by evidence-based guidelines published by WHO [2, 5, 25]. In this study hand hygiene was assessed using Hand Hygiene Audit Checklist, a widely used to assess health professionals’ compliance with hand hygiene [20]. The checklist assesses compliance with hand hygiene in the five opportunities, indications, or “moments” recommended by the WHO and the action taken, with three possibilities of: (1) rubbed with alcohol; (2) washed with water and soap; (3) not performed. Option 3 includes using gloves instead of performing hand hygiene [20].

Data were collected before the intervention and 6 months after. The period of 6 months chosen to evaluate after the intervention, due to the time constraints of this study (it was a master’s thesis). Nurses in intervention and control group were invited to complete a paper-based survey including questions about sociodemographic characteristics and work-related factors, and the CSPS.

Direct observation of healthcare workers during patient care activities by trained and validated observers is recognized as the gold standard for hand hygiene monitoring (Sax, Allegranzi et al. 2009). In this study, data on hand hygiene compliance were collected before and 6 months after the intervention by a trained observer (ShGM), who was also a member of the research team. A non-participant direct observation was conducted. The timings of the observation sessions were randomly distributed throughout the week days. The observer researcher registered the opportunity for hand hygiene and whether hand hygiene was performed, in accordance with the WHO’s five moments for hand hygiene.

### Development of the ICLNs program

The researchers developed the ICLNs program following a comprehensive review of literature on the ICLN programs and identifying factors that contributed to the success of the programs in acute care settings [14, 15, 26–29]. Previous studies suggest that the success of the ICLN program depends to a great deal on identifying and preparing right ICLNs, and the support available for them [14]. Education, commitment, and coordination by the IPC team, support from the ward management, support from the senior hospital management, and peer support are essential and should be considered in developing ICLN programs [14].

In the current study, two ICLNs were selected from each participating ward in the intervention group. They received training with the aim of promoting standard precautions and hand hygiene within their ward. The research team (ShSh and ShGM) conducted the training of the ICLNs with cooperation of the hospital’s infection control nurse and the educational supervisor. The training included reviewing the guidelines on standard precaution measures and hand hygiene and discussing the rationale for maintaining an optimal level of compliance with standard precautions and hand hygiene. The ICLNs also received a hard copy of the educational materials for future reference. They worked closely with the infection control nurse of the hospital and attended monthly meetings with the research team. They educated staff in their ward about infection control and encouraged them to comply with ensuring compliance with infection control guidelines promoted compliance with infection control guidelines (e.g. hand hygiene and personal protective equipment). The role of the head nurses were to support the ICLNs and consider and address any critical organizational problems reported by the ICLNs.

### Training of observer

Before commencing actual observations, the observer was trained and tested in assessing compliance with hand hygiene according to the observation guidelines of WHO. Training included watching an educational video of healthcare workers performing patient care tasks and listening to several educational presentations [30]. Then, the observer was engaged in inter-rater reliability testing, in which a series of hand hygiene practices were co-assessed by the observer and another member of the research team (ShSh), and disagreements were discussed and resolved according to WHO hand hygiene training tools [30]. In addition, two assessors performed assessments on randomly selected subset of observation sessions. The inter-rater reliabilities, using Kappa coefficients, for these sessions ranged from + 0.62 to + 1, indicating a good- to- very good inter-rater agreement [31, 32].

### Sample size

In order to determine the sample size for compliance with Standard Precautions variable in this study and to calculate effect size, the primary information including mean and standard deviation of compliance with the Standard Precautions was derived from Donati et al.’s study [13]. Considering a two-sided 5% significance level and a power of 80%, a sample size of 77 participants per group was necessary.

Sample size for hand hygiene observations was determined based on the WHO Hand Hygiene Technical Reference Manual, which suggests 200 opportunities per unit per observation period [20]. Considering this recommendation, a sample size of 1600 opportunities per observation period per group was considered necessary.

### Data analysis

Analyses were done with the Statistical Package for Social Sciences (IBM SPSS software (version 26; SPSS, Chicago, IL). Two independent sample t-tests were used to examine differences between hand hygiene practices among nurses in intervention and control group. Multiple linear regression analysis was used to assess the effect size. All *p* values were based on two-tailed tests, with statistically significance defined as *p <* 0·05.

## Results

### Participant characteristics

Data were collected from all 16 participating hospital wards. A total of 154 clinical nurses participated in the study; 77 nurses from each study group. All the nurses completed the CSPS. Participants were mainly female (76.4%) with average age of 30.20 ± 5.32 years, and had an average 6.55 ± 4.94 years of clinical nursing experience. The average nurse-to-patient ratio was 1.7 nurse to 10 patients, and average working hours per week was 46.43 ± 5.34 h. Only 57.8% of participants had completed a training course on standard precautions and 63.6% on hand hygiene previously. The demographic and clinical characteristics of participants are shown in Table 1. Between-group differences in demographic and professional characteristics were not statistically significant (Table 1).

**Table 1:** Baseline demographic and clinical characteristics of participants in the ICLN Program (N = 154)

Variables	Categories	Total n (%) (n = 154)	Intervention n (%) (n = 77)	Control n (%) (n = 77)	*p*-Value
**Gender**	Female	117 (76)	56(72.7)	61(79.2)	0.346^a^
	Male	37 (24)	21(27.3)	16(20.8)	
	Total	154 (154)	77(100)	77(100)	
**Age (years)**	Mean ± SD	30.20 ± 5.32	29.87 ± 5.40	30.54 ± 5.26	0.550^b^
**Education**	Bachelor degree	144 (93.5)	74 (96.1)	70 (90.9)	0.327^b^
	Master’s degree	9 (5.8)	3. (3.9)	6 (7.8)	
	Total	153(99.4)	77(100)	76(98.7)	
**Marital status**	Married	99(64.3)	50(64.9)	49(63.6)	0.866^a^
	Single/Widow/Separation	55(35.7)	27(35.1)	28(36.4)	
**Clinical experience (years)**	Mean ± SD	6.55 ± 4.94	6.54 ± 5.12	6.52 ± 4.80	0.979^c^
**Training about standard precautions at the hospital**	yes	89 (57.8)	42(54.5)	47(60.1)	0.116^c^
	Mean ± SD	7.6 ± 8.72	6.07 ± 4.89	9.00 ± 11.00	
**last time from the training**	Mean ± SD	2.07 ± 1.87	1.68 ± 2.04	3.65 ± 4.89	0.065^c^
**Hand hygiene training sessions**	1–2	72 (46.8)	30(39.0)	42(54.5)	0.285^a^
	3 and greater	26 (16.9)	14(18.2)	12(15.6)	
	Total	98(63.6)	44(57.1)	54 (70.1)	

^a^Chi squared test; ^b^Chi-square Monte Carlo simulation test; ^c^ The independent samples t-test

### Compliance with standard precautions

Table 2 summarizes data on participants’ self-reported compliance with standard precaution measures. In intervention group, compliance with standard precautions increased slightly from pre-test (13.37 ± 3.33) to post-test (14.03 ± 3.64), however, the pre-post intra-group difference was not statistically significant (*p* = 0.076). In control group, compliance with standard precautions decreased slightly from pre-test (12.72 ± 4.00) to post-test (12.59 ± 4.54), with no statistically significant pre-post intra-group difference (*p* = 0.781). The baseline assessment did not reveal a statistically significant difference between intervention and control group in compliance with standard precautions (13.37 ± 3.33 vs.12.72 ± 4.00, p = 0.276). The post-test difference between the groups was not also statistically significant (β = 5.18; 95% CI-0.3-10.65, p = 0.064).

**Table 2:** Comparison of compliance with standards precautions between the intervention and the control group in the ICLN Program (n = 154)

Variable	Group	Pretest					Posttest					p-Value	p-Value	p-Value®
		Compliance with SPs, %				Overall Compliance Rate %	Compliance with SPs, %				Overall Compliance Rate %			
		Never	Seldom	Sometimes	Always		Never	Seldom	Sometimes	Always				
I wash my hands between patient contacts	Intervention	1.3	7.8	19.5	71.4	71.4	0.0	1.3	5.2	93.5	93.5	< 0.001	0.005	< 0.001
	Control	0.0	2.6	33.8	63.6	63.6	0.0	2.6	28.6	68.8	68.8	0.556		
p-value						0.302^*^					< 0.001^*^			
I only use water for hand washing	Intervention	27.3	10.4	53.2	9.1	27.3	27.3	18.2	42.9	11.7	27.3	0.999	0.496	0.906
	Control	37.7	11.7	40.3	10.4	37.7	29.9	24.7	33.8	11.7	29.9	0.327		
p-value						0.169^*^					0.721^*^			
I use alcohol hand rubs as an alternative if my hands are not visibly soiled	Intervention	9.1	5.2	41.6	44.2	44.2	1.3	5.2	59.9	41.6	41.6	0.845	0.568	0.371
	Control	7.8	6.5	49.4	36.4	36.4	6.5	11.7	49.4	32.5	32.5	0.678		
p-value						0.324^*^					0.243^*^			
I recap used needles after giving an injection	Intervention	51.9	24.7	18.2	5.2	51.9	58.4	27.3	13.0	1.3	58.4	0.383	0.045	0.806
	Control	39.0	24.7	24.7	11.7	39.0	50.6	31.2	11.7	6.5	50.6	0.081		
p-value						0.106^*^					0.332^*^			
I put used sharp articles into sharps boxes	Intervention	3.9	0.0	2.6	93.5	93.5	0.0	0.0	2.6	97.4	97.4	0.450	0.789	0.160
	Control	0.0	0.0	6.5	93.5	93.5	0.0	3.9	3.9	92.2	92.2	0.999		
p-value						0.999^**^					0.276^**^			
The sharps box is only disposed when it is full	Intervention	53.2	19.5	16.9	10.4	53.2	62.3	13.0	14.3	10.4	62.3	0.211	0.522	0.017
	Control	42.9	19.5	23.4	14.3	42.9	40.3	22.1	24.7	13.0	40.3	0.803		
p-value						0.197^*^					0.006^*^			
I remove PPE in a designated area	Intervention	1.3	2.6	22.1	74.0	74.0	1.3	9.1	7.8	81.8	81.8	0.211	0.499	0.028
	Control	1.3	6.5	27.3	64.9	64.9	1.3	6.5	28.6	63.6	63.6	0.999		
p-value						0.221^*^					0.011^*^			
I take a shower in case of extensive splashing even after I have put on PPE	Intervention	11.7	7.8	28.6	51.9	51.9	3.9	13.0	24.7	58.4	58.4	0.458	0.212	0.907
	Control	13.0	15.6	20.8	50.6	50.6	9.1	14.3	19.5	57.1	57.1	0.404		
p-value						0.872^*^					0.870^*^			
I cover my wound (s) or lesion (s) with waterproof dressing before patient contacts	Intervention	3.9	20.8	23.4	51.9	51.9	2.6	18.2	23.4	55.8	55.8	0.677	0.671	0.246
	Control	5.2	10.4	26.0	58.4	58.4	7.8	10.4	32.5	49.4	49.4	0.248		
p-value						0.418^*^					0.420^*^			
I wear gloves when I am exposed to body fluids, blood products, and any excretion of patients	Intervention	0.0	1.3	13.0	85.7	85.7	0.0	5.2	13.0	81.8	81.8	0.579	0.556	0.940
	Control	0.0	2.6	15.6	81.8	81.8	0.0	2.6	16.9	80.5	80.5	0.999		
p-value						0.512^*^					0.837^*^			
I change gloves between each patient contact	Intervention	1.3	9.1	27.3	62.3	62.3	0.0	3.9	18.2	77.9	77.9	0.038	0.045	0.147
	Control	1.3	2.6	28.6	67.5	67.5	0.0	3.9	26.0	70.1	70.1	0.789		
p-value						0.499^*^					0.270^*^			
I decontaminate my hands immediately after removal of gloves	Intervention	0.0	1.3	20.8	77.9	77.9	0.0	1.3	15.6	83.1	83.1	0.502	0.499	0.084
	Control	0.0	5.2	28.6	66.2	66.2	0.0	9.1	23.4	67.5	67.5	0.999		
p-value						0.106^*^					0.025^*^			
I wear a surgical mask alone or in combination with goggles, face shield, and apron whenever there is a possibility of a splash or splatter	Intervention	0.0	2.6	24.7	72.7	72.7	0.0	1.3	32.5	66.2	66.2	0.359	0.651	0.809
	Control	2.6	6.5	33.8	57.1	57.1	0.0	3.9	37.7	58.4	58.4	0.999		
p-value						0.043^*^					0.318^*^			
My mouth and nose are covered when I wear a mask	Intervention	0.0	1.3	10.4	88.3	88.3	1.3	1.3	13.0	84.4	84.4	0.606	0.186	0.556
	Control	2.5	2.5	7.6	87.0	87.0	1.3	5.2	13.0	80.5	80.5	0.267		
p-value						0.806^*^					0.525^*^			
I reuse mask or disposable PPE	Intervention	53.2	13.0	13.0	20.8	53.2	59.7	15.6	19.5	5.2	59.7	0.441	0.142	0.781
	Control	46.8	11.7	20.8	20.8	46.8	55.8	16.9	14.3	13.0	55.8	0.265		
p-value						0.420^*^					0.625^*^			
I wear a gown or apron when exposed to blood, body fluids, or any patient excretions	Intervention	1.3	5.2	24.7	68.8	68.8	1.3	13.0	23.4	62.3	62.3	0.424	0.551	0.819
	Control	2.6	1.3	35.1	61.0	61.0	1.3	6.5	31.2	61.0	61.0	0.999		
p-value						0.311^*^					0.868^*^			
Waste contaminated with blood, body fluids, secretion, and excretion are placed in red plastic bags irrespective of patient’s infective status	Intervention	0.0	2.6	5.2	92.9	92.9	0.0	0.0	9.1	90.9	90.9	0.999	0.136	0.085
	Control	5.2	0.0	5.2	89.6	89.6	0.0	3.9	15.6	80.5	80.5	0.121		
p-value						0.575^*^					0.065^*^			
I decontaminate surfaces and equipment after use	Intervention	2.6	7.8	35.1	54.5	54.5	1.3	2.6	33.8	62.3	62.3	0.345	0.755	0.212
	Control	5.2	6.5	28.6	59.7	59.7	1.3	9.1	33.8	55.8	55.8	0.579		
p-value						0.515^*^					0.413^*^			
I wear gloves to decontaminate used equipment with visible soils	Intervention	0.0	1.3	13.0	85.7	85.7	0.0	2.6	10.4	87.0	87.0	0.999	0.999	0.391
	Control	0.0	3.9	11.7	84.4	84.4	0.0	3.9	14.3	81.8	81.8	0.814		
p-value						0.821^*^					0.374^*^			
I clean up spillage of blood or other body fluid immediately with disinfectants	Intervention	5.2	3.9	14.3	76.6	76.6	3.9	6.5	18.2	71.4	71.4	0.502	0.499	0.157
	Control	3.9	1.3	10.4	84.8	84.8	1.3	5.2	10.4	83.1	83.1	0.999		
p-value						0.222^*^					0.084^*^			
Intervention group					66.8					70.1	0.149	0.098		0.064
Control group					63.6					62.9	0.450			
					0.059^**^					0.019^**^				

* chi-square

The item compliance rate refers to the mean score of each item.

**Monte Carlo Simulation

ΦMcNemar’s test

®Binery logistic regression

However, there was seen a statistically significant difference in the dimension of ‘cross infection from person to person’ (β = 8.48, CI 95%=1.71 to 15.26, p-value = 0.014) between the groups. Table 3 demonstrates the comparison of mean scores of different dimensions of the CSPS between the groups. Also, analyzing data specifically for Item I ‘I wash my hands between patient contacts’ showed a statistically significant difference between the groups (p < 0.001).

**Table 3:** The comparison of mean scores of different dimensions of the CSPS between groups in the ICLN Program (N = 154)

Variable	Group	Pre	post	^**^P-Value	β (95% CI)	^***^P
		Mean ± SD	Mean ± SD			
**Use of protective device: (Items 7,10,13,14,15,16)**						
Total score	I	4.42 ± 1.45	4.36 ± 1.57	0.710	1.91 (-6.15 to 9.98)	0.640
% Score		73.8	72.7			
Total score	C	3.98 ± 1.63	4.00 ± 1.88	0.948		
%Score		66.4	66.6			
^*^ **P-Value**		0.079	0.196			
**Disposal of sharps: (Items 4,5,6)**						
Total score	I	1.98 ± 0.85	2.18 ± 0.72	0.050	8.05 (0.50 to 15.61)	0.037
%Score		66.2	72.7			
Total score	C	1.75 ± 0.82	1.83 ± 0.87	0.400		
%Score		58.4	61.0			
^*^ **P-Value**		0.086	0.008			
**Disposal of waste (Item 17)**						
Total score	I	0.92 ± 0.26	0.90 ± 0.28	0.708	9.38 (-1.16 to 19.94)	0.081
%Score		92.2	90.9			
Total score	C	0.89 ± 0.30	0.80 ± 0.39	0.070		
%Score		89.6	80.5			
^*^ **P-Value**		0.578	0.066			
**Decontamination of spills and used article (Items 18,19,20)**						
Total score	I	2.16 ± 0.90	2.20 ± 0.96	0.741	1.89 (-7.59 to 11.39)	0.693
%Score		72.2	73.5			
Total score	C	2.28 ± 0.88	2.20 ± 0.01	0.483		
%Score		76.1	73.5			
^*^ **P-Value**		0.420	0.999			
**Prevention of cross infection from person to person (Items 1,2,3,8,9,11,12)**						
Total score	I	3.87 ± 1.46	4.37 ± 1.43	0.009	**8.48** **(1.71 to 15.26)**	**0.014**
%Score		55.2	62.5			
Total score	C	3.80 ± 1.73	3.75 ± 1.84	0.799		
%Score		54.3	53.6			
^*^ **P-Value**		0.802	0.020			
**Overall Compliance with Standard Precautions**						
Total score	I	13.37 ± 3.33	14.03 ± 3.64	0.076	5.18 (-0.3 to 10.65)	0.064
%Score		66.8	70.1			
Total score	C	12.72 ± 4.00	12.59 ± 4.54	0.781		
%Score		63.6	62.9			
^*^ **P-Value**		0.276	0.031			

ICLN, Infection control link nurse program; I,Intervention Group; C,Control Group;

*, based on independent t-test; **, based on paired Sample t-test; ^***^ based on Multiple linear regression with control of the effect of basic variables

### Compliance with hand hygiene

Regarding the compliance with hand hygiene, a total of 6868 opportunities for hand hygiene were observed over 382 sessions (an average of 17 opportunities per session). During the pre-test period (from June 1, 2021 to July 18, 2021; 50 days), 3431 hand hygiene opportunities were recorded from 191 observation sessions, while in the post-test period (from February 8, 2022, to March 13, 2022; 34 days), 3437 hand hygiene opportunities were recorded from 191 observation sessions.

In pre-test, participants in intervention and control groups performed only 18.80% and 16.48% of the hand hygiene opportunities, respectively. In other words, participants in intervention group missed 81.2% and control group 83.52% of the hand hygiene opportunities, with no statistically significant difference between the groups (*p* = 0.264). In 29.47% of the missed opportunities, participants in intervention group used gloves instead of hand hygiene compared with 26.22% in control group, with no between-group difference in use of gloves at baseline (*p =* 0.995).

In post-test, participants in intervention group and control group performed hand hygiene in 37.32% and 16.18% of the hand hygiene opportunities, respectively. In other words, participants in intervention group and control group missed 62.68% and 83.82% of the hand hygiene opportunities, respectively, with a statistically significant between-group difference (*p* < 0.001). Of the missed opportunities, 15.01% and 24.30% were due to using gloves instead of hand hygiene in intervention and control groups, respectively, with a statistically significant between-group difference in use of gloves post intervention (*p =* 0.011). Comparing the pre-post intra-group differences, compliance with hand hygiene improved by 18.52% in intervention group (*p* < 0.001) compared with 0.48% in control group (*p* = 0.765). Comparing the pre-post intra-group differences, use of gloves instead of hand hygiene decreased by 14.38% in intervention group (*p =* 0.001) compared with 1.97% in control group (*p =* 0.225) (Table 4).

**Table 4:** Number of hand hygiene opportunities, actions not performed, frequencies and proportions of glove use in the HH moments (n = 6868 observations)

Group	Pretest			Posttest			*p* value ^b^	*P v*alue ^c^
	Hand hygiene Opportunities	Hand hygiene not performed	Frequency of glove use	Hand hygiene Opportunities	Hand hygiene not performed	Frequency of glove use		
Intervention	1719	1391	410	1701	1047	158	< 0.001	< 0.001
Control	1712	1430	375	1736	1443	350	0.765	
*p* value ^a^		*p* = 0.264	*p* = 0.995		*p* < 0.001	*p = 0.011*		

^a^ Independent Samples T Test; ^b^ Paired Samples T Test; ^c^ Multiple linear regression with baseline scores controlled

There were statistically significant differences between intervention group and control group in compliance with hand hygiene in all the hand hygiene moments including before patient contact (*p* = 0.002), before performing an aseptic task (*p* = 0.002), after body fluid exposure (*p* = 0.006), after patient contact (*p* < 0.001), and after contact with patient surroundings (*p* < 0.001). The main improvement was recorded in compliance with the moment 5 (after touching patient surroundings), which increased from 23.03 to 45.74% in the intervention group.

## Discussion

The purpose of this research was to examine the effectiveness of an infection control link nurse (ICLN) program on compliance with standard precaution measures and hand hygiene among nurses. Overall, compliance with standard precautions among the participant nurses in both intervention and control group at pre and post intervention phases was low and in the range of “below the optimal level”. These findings are in agreement with some previous studies, reporting compliance with standard precautions among hospital nurses as suboptimal, with no significant improvements after interventions [33, 34]. This finding is; however, incongruent with that of Donati et al. (2020) in Italy, which found that nurses in both groups reported significantly increased compliance with standard precaution scores after intervention compared to baseline, with greater increase being observed in intervention group [13]. These inconsistencies could be attributed to differences in the ICLN programs across the studies. The self-report nature of the Compliance with Standard Precautions Scale and cultural differences in using these types of scales may have contributed to the nonsignificant result [35, 36]. In addition, infection control link nurses’ commitment to the role, the support they received from the infection control nurse, their managers and colleagues, and time release to complete the role can be factors affecting the effectiveness of ICLN program on nurses’ compliance with SPs [8, 37, 38]. The effects of these factors on the success of the ICLN programs should be investigated in future research.

In pre-test, participants in intervention and control groups performed hand hygiene in only 18.80% and 16.48% of the existing hand hygiene opportunities, respectively. This low level of compliance with hand hygiene among nurses in current study is concerning and needs a close attention of the health authorities to improve hand hygiene performance, as the cornerstone of HCAIs preventative measures, among health care providers by implementing effective evidence based strategies. This finding is in line with several previous studies, reporting suboptimal hand hygiene compliance among nurses [39]. In a study by Ataiyero et al. (2022) in Nigeria, hand hygiene compliance was 29.1% among health care workers in surgical wards [37] In another study by Oyibo et al. (2022) in Nigeria, the covertly observed compliance rate was fund to be 18.6% [39]. However, our finding is incongruent with the result of systematic review which reported hand hygiene compliance among nurses in Iran to be 40.5% [40]. A reasonable explanation of why the hand hygiene compliance results in the present study is differ from the results of the systematic review from the country may be due to the heterogeneity among studies included in their final analysis in terms of the measurement instrument (the WHO instrument and others), the source of reporting the adherence (observation vs. self-reporting), and the unit of measurement (person vs. opportunity) as have mentioned by the authors as their study limitations [40].

In an interventional study conducted by Donati et al. (2020) in Italy, baseline hand-hygiene compliance rates among nurses in control and intervention groups were 63% and 61.9% [13].

In current study, the ICLN program was effective to significantly improve compliance with hand hygiene among participants in intervention group. Participants in intervention group reported an increase of 18.52% in hand hygiene performance post intervention. This is a significant improvement in hand hygiene not only statistically but also clinically. Close to 20% to indicate a clinical significance. This finding is in line with previous similar studies which evaluated the effectiveness of ICLN programs. In a study conducted by Donati et al. (2020) in Italy, nurses in the ICLN group reported an increase of 14.3% in hand hygiene compliance [13]. The finding of this study confirmed the effectiveness ICLN programs in improving hand hygiene compliance. Improvements were observed in all hand hygiene moments, however, the greatest improvement occurred in the moment 5, which is performing hand hygiene after touching a patient’s surroundings. In Donati et al. study (2020), observed compliance with the first moment “before touching a patient” had the greatest increase (Donati, Miccoli et al. 2020).

A reasonable explanation of this finding is promoting hand hygiene practices by ICLN nurses who acted as role models and trained and influenced hand hygiene practices of other nurses in their wards.

The ICLN program in this study was also effective in reducing the inappropriate use of gloves as an alternative for hand hygiene by 14.38%. Improvements in hand hygiene performance and reduction in the use of gloves an alternative to hand hygiene are significant findings, indicating the important role of infection control link nurses role in improving hand hygiene practice of nurses in the clinical settings.

### Study strengths and limitations

There are a number of strengths associated with this research study. First, direct observation was used to collect the HHC data, which is considered the ‘gold standard’ method of measuring HH compliance [41]. The findings added to our understanding of the effectiveness of ICLN programs on improving the HH compliance. Nevertheless, the study has some limitations to consider. Direct observation method has a few limitations. It is timeconsuming, it requires a dedicated trained staff, and there is a chance of observation bias - the Hawthorne effect. The ones being observed may have improved their hand hygiene compliance, because someone was watching them [42]. However, in the study hospital, we do not have electronic soap dispensers/electronic surveillance technology. Therefore, a singleobserver direct observation technique was used and compliance with HH was evaluated. The outcome assessor was aware of the group allocations, which may introduce some bias to the study, although the observer was trained and tested in assessing compliance with hand hygiene according to the observation guidelines of WHO before the commencement of the study. Also, allocating wards to intervention and control groups in one single hospital may introduce contamination bias; although this was less likely due to the very large size of the hospital. Finally, nurses were recruited from different wards of only one hospital; therefore, the findings could be generalizable only to settings that have similar characteristics to those of this study. Separating the study group’s geographically in a multicenter study may improve the findings. Further research is necessary to validate our study findings using structured research programs in this area.

## Conclusions

To the best of our knowledge, this is the first study that investigated the effectiveness of the implementing an infection control link nurse program (ICLN) on compliance with standard precautions and hand hygiene compliance of nurses in Iran. In this study, compliance with standard precautions was in the range of “below the optimal level” and implementation of the infection control link nurse program had no statistically significant effect on improving the compliance with standard precautions of the studied nurses. However, the infection control link nurse program was effective in improving hand hygiene compliance of nurses. Given the continuing level of interest that exists in improving health care workers’ hand hygiene practices, the findings of this study provide significant practical implications for hospitals seeking to improve compliance with hand hygiene among nurses, showing the effectiveness of using infection control link nurse program. Further research is needed to assess effectiveness of using infection control link nurse program to improve compliance with standard precautions and the long term effects of the ICLN programs.

## Data Availability

The datasets generated and analyzed during the current study are not publicly available due to the confidentiality of the participants but are available from the corresponding author on reasonable request.

## Abbreviations

IPC: Infection Prevention and Control
HCAIs: Healthcare-associated Infections
HCWs: Healthcare Workers
WHO: World Health Organization
HHC: Hand hygiene compliance
ICLN: Infection control link nurse program
